# Predicting Engagement and Retention During an Online Theory‐Based Intervention to Promote Physical Activity Among Inactive Parent‐Child Dyads

**DOI:** 10.1002/ejsc.70060

**Published:** 2025-10-08

**Authors:** Daniel J. Phipps, Weldon T. Green, Taru Lintunen, Martin S. Hagger, Keegan Knittle

**Affiliations:** ^1^ Faculty of Sport and Health Sciences University of Jyväskyla Jyväskylä Finland; ^2^ School of Applied Psychology Griffith University Brisbane Australia; ^3^ Department of Psychological Sciences, Health Sciences Research Institute University of California—Merced Merced California USA

**Keywords:** intervention adherence, intervention fidelity, motivation, physical activity, psychology

## Abstract

Insufficient physical activity is a widespread health concern, necessitating the broad implementation of evidence‐based behavior change interventions. Such evidence commonly derives from randomized controlled trials, but questions arise about who is willing to enroll and actively engage in such trials. This study investigated factors predicting engagement and retention in an online physical activity intervention for inactive parent‐child dyads. Participants were recruited from the general Finnish population and assigned to either an intervention or wait‐list control group. The intervention consisted of online materials, SMS prompts, and four online sessions. Partial least squares regression models were used to analyze autonomous motivation, parent and child gender, parental education, employment status, and recruitment source as predictors of intervention retention and engagement. Results showed that intervention retention was predicted by higher autonomous motivation, being a mother, social media recruitment, and university education. Session attendance was higher for autonomously motivated parents, fathers, parents of daughters, and university‐educated parents. These findings highlight the importance of autonomous motivation and demographic factors in intervention engagement. However, the higher engagement of already motivated participants demonstrates challenges for reaching and intervening on those who might benefit most from such programs. Future research should explore strategies to engage and retain less motivated individuals and investigate reasons behind non‐compliance to improve the effectiveness of physical activity interventions for inactive families.

## Highlights

- A key challenge for successful behavior change in physical activity is engaging and retaining in behavior change programs those who are most in need of such programs but might otherwise be unmotivated to participate.
- In a Finnish parent–child physical activity trial, retention was higher among autonomously motivated, university‐educated parents, mothers, and those recruited via social media.
- Engagement patterns differed: session attendance was higher for autonomously motivated, university‐educated parents, fathers, and parents of daughters, indicating distinct determinants for attending sessions versus remaining to complete outcome measures.

## 1 Introduction

Insufficient levels of physical activity are associated with numerous negative health consequences and lower quality of life (Åman et al. 2009; Iso‐Markku et al. 2024), while engaging in regular physical activity is associated with improvements in psychological wellbeing and a reduced risk of chronic health conditions (Haskell et al. 2009). Yet, despite strong evidence on the value of engaging in an active lifestyle, most people in many developed countries still fall short of recommended levels of physical activity (Wennman and Borodulin 2021). Considering the physical activity deficits across the population, a key focus by many governments and health groups has been the development and implementation of intervention strategies to promote an active lifestyle or reduce sedentary behavior. However, to make firm and meaningful conclusions on the efficacy of such strategies, it is important that they undergo rigorous, theory‐based development and evaluation, necessitating the use of randomized controlled trials as part of the behavior change process. One such example of a physical activity based randomized controlled trial is the ProAct project (Phipps et al. 2024)—an online randomized controlled trial to improve activity levels in inactive parent‐child dyads based on the integrated behavior change model (Hagger and Chatzisarantis 2014).

While randomized controlled trials like the aforementioned ProAct trial are generally accepted as the gold‐standard of behavior change evidence and a required element of wider public health strategy development, they carry several pitfalls from a scientific perspective. Foremost among these is the long‐standing issue of recruiting and retaining samples in psychological research (Krusche et al. 2014; Leslie et al. 2022). That is, a key issue in psychological research is that engaging in intervention research is generally an involved process, which inherently requires some motivation to act. To some degree, researchers may be able to influence this motivation, for example by providing monetary rewards, prizes, or compensation (Linardon and Fuller‐Tyszkiewicz 2020). However, when considering the need to scale interventions for effective population level change, such incentive‐based, external motivations for compliance rapidly become unfeasible and costly. Instead, to investigate the more natural or realistic effects of an intervention and who is willing to engage in it, it is vital to investigate not only the effectiveness of an intervention, but also its compliance, in the absence of extraneous motivators.

### 1.1 Predicting Intervention Compliance

In terms of previous health behavior change interventions, several dispositional and motivational factors have been identified as potential correlates of intervention fidelity among participants. For example, women and people with higher education backgrounds have been suggested to have better adherence and retention to longitudinal research and intervention programs (Beatty and Binnion 2016; Rose et al. 2024; J. Ryan et al. 2017), albeit with generally small effects. Busy lifestyles are also frequently reported as a key reason for intervention non‐adherence or dropout in psychological research (Beatty and Binnion 2016). However, it is also important to consider that such findings regarding the structural and demographic correlates of intervention fidelity stem from wider research in health, and the current context, a family based physical activity intervention, may have unique traits regarding demographic effects. For example, while women are generally more engaged in health interventions, fathers have been shown to be particularly engaged and influential in their children's physical activity and sport (Knoester and Randolph 2019; Morgan et al. 2019; Schoeppe et al. 2016).

It is also plausible that the motivations present in samples prior to enrolling in the intervention affect intervention fidelity. For example, an RCT encouraging safe sex found that those who already had more knowledge about sexually transmitted diseases were more likely to complete and engage in the intervention program (DiFranceisco et al. 1998), while a voluntary program encouraging water safety behavior for parents found that those who enrolled in the trial reported very high levels of motivation and attitude in favor of the target behavior before the intervention began (Hamilton et al. 2019). Some researchers have attempted to address this, creating metrics of potential for change in the key psychological determinants of health behavior (Knittle and Peters 2019). However, even adjusting for how potentially high levels of motivation at baseline might skew results, it is important to consider how pre‐existing motivations might affect intervention adherence and completion overall.

One key finding in regard to persistence in general has been around motivational quality, as drawn from self‐determination theory (Deci and Ryan 1985). That is, when an individual is autonomously motivated toward a behavior (i.e., believes engaging in a behavior would be enjoyable or consistent with their core values), they are more likely to engage in that behavior over time (Rottensteiner et al. 2015; Phipps et al. 2022), even when facing barriers or challenges to doing so. Given its theoretical impact on long term behavior, autonomous motivation represents a key target construct for many psychological behavior change interventions. However, given its relationship to engagement and persistence (Rottensteiner et al. 2015; Azila‐Gbettor et al. 2021; Vansteenkiste et al. 2004), it is also plausible that having autonomous motivation at baseline may also be linked to engaging and completing an intervention, although evidence specifically testing this is sparce. Indeed, in most social cognitive and motivation research, the use of autonomous motivation and similar constructs follows the principle of compatibility (Ajzen 1991; Ajzen and Fishbein 1977). That is, where the target behavior or outcome strongly aligns with the measures of motivation and social cognitive beliefs, for example with levels of moderate‐to‐vigorous physical activity predicted by scores on measures of autonomous motivation toward engaging in moderate‐to‐vigorous physical activity. However, in the current study, we apply the autonomous motivation for physical activity construct alongside demographic factors to predict not the engagement in physical activity itself, but the engagement with an intervention aiming to promote physical activity by targeting the constructs of the integrated behavior change model. Such a supposition assumes that enrolling in an intervention represents a degree of willingness to change one's behavior. However, even if all participants in the current intervention are at least somewhat motivated to change, it might also be assumed that the motivations and beliefs of the sample about physical activity at baseline may also influence how likely they are to complete their intervention program successfully.

### 1.2 The Current Study

In the current study we aimed to test how autonomous motivation, alongside demographic factors (parent gender, child gender, employment status, parental education), predicted the completion of, and engagement with, a theory‐based intervention to encourage physical activity. Specifically, the current research is part of the wider ProAct project (Phipps et al. 2024)—an intervention based on promoting physical activity in insufficiently active parent‐child dyads by improving their autonomous motivations, beliefs, and habits around being active. In the current study, we predicted that, as per self‐determination theory, autonomous motivation would predict intervention engagement and completion. We also predicted that higher intervention completion and engagement when dyads comprised of fathers or sons, when parents were not currently employed, and when parents were university educated.

## 2 Method and Materials

### 2.1 Participants and Procedures

Participants in the current study were part of the wider ProAct project, an intervention aiming to improve physical activity levels in inactive parent‐child dyads recruited from the general Finnish population. In the current research, participants were recruited either via a panel company or via advertisements on social media and were eligible to participate in the research if parents were not active for at least 30 min a day on five or more days in the past week and children were not active for at least 60 min per day in the past week. In total, 88 participants enrolled in the trial and provided baseline data. Once participants were enrolled into the trial and assigned to either the intervention or wait‐list control group, they received no incentives for their continued participation, other than the right to access to materials of the program itself. The current study included only those who passed screening requirements and completed the baseline survey. As part of the program, all participants, regardless of assignment to the intervention or waitlist control group, were asked to complete survey measures at signup, 3‐month post‐signup, and 6‐month post signup. A full description of study procedures and the intervention tasks can be accessed via the study protocol (Phipps et al. 2024). As retainment and intervention engagement were our outcome measures, we experienced no attrition in regard to follow‐up outcome assessment. All procedures were approved by the University of Jyväskylä Human Sciences Ethics Committee.

### 2.2 The ProAct Intervention

The ProAct intervention was assessed in a randomized controlled trial, where participants were assigned to either an immediate intervention group or a waitlist control group. The ProAct intervention consisted of online materials, SMS based prompts, and four online sessions hosted via the Zoom platform. Parents were invited to attend all four sessions and their child was invited to attend sessions 2–4 (i.e., not session 1).

As part of our steps for encouraging participant engagement, all participants received a phone call after sign‐up, allowing them to ask questions about the study, informing them to expect survey measures via email, and asking participants assigned to the intervention group to enlist in intervention sessions. When participants were signed up to sessions, they received three email reminders in the week leading up to each session, plus an SMS text message reminding them of the session on the day. Similarly, for each data collection point, participants were emailed links to online surveys to complete and called to confirm the details of data collection. Where calls and emails went unanswered, follow‐up emails and text messages and calls were attempted to re‐establish contact.

### 2.3 Measures

#### 2.3.1 Intervention Retention and Engagement

Dropout from the intervention was assessed both through specifically notifying the research team that participants did not wish to participate further, or through non‐response to contact attempts and data collection. Where participants stayed in the intervention program and provided any follow‐up measures, they were marked as retained = 1, while participants who dropped out of the intervention either by explicit request or non‐response were marked as 0.

#### 2.3.2 Session Attendance

Parents attendance at each session was assessed following the inspection of Zoom logs for each intervention meeting. Summing these gave each parent in the intervention group a total score of sessions attended, which could range from zero to four.

#### 2.3.3 Autonomous Motivation

Autonomous Motivation for physical activity was assessed in parents using four items with the common stem “I (would) do physical activity during my free time…” (e.g., “… because I enjoy doing physical activity”) (R. M. Ryan and Connell 1989). Responses were provided on five‐point Likert scales (1 = strongly disagree to 5 = strongly agree). The scale displayed good internal consistency (*α* = 0.81).

#### 2.3.4 Participant Demographics

Parents in each dyad were asked to report on their demographic information. Demographic groups were then operationalized as dummy coded variables for parent gender (male = 1, female = 0), child gender (male = 1, female = 0), parents' education (university educated = 1, non‐university educated = 0), and parents' employment status (employed = 1, unemployed or stay‐at‐home parent = 0).

#### 2.3.5 Source of Recruitment

As a covariate in analysis, participants recruitment source was included in analyses as a dummy coded variable such that participants recruited from social media were assigned a score of 1, while participants recruited from a panel company were assigned a score of 0.

### 2.4 Data Analysis

Data were analyzed using partial least squares regression modeling via the WarpPLS 8.0 software, given the generally robust nature of PLS modeling with smaller sample sizes and non‐normal distributions (Hair and Alamer 2022). Autonomous motivation was included as a linear composite, while all other variables were included as dummy‐coded dichotomous variables. Standard errors and *p* values were calculated via default recommended WarpPLS8.0 settings ‐ the stable3 method with one‐tailed tests (Kock 2022). Model quality was assessed using the Tennenhaus goodness‐of‐fit index (GoF, acceptable if > 0.1 when effect sizes are small, or > 0.25 if effect sizes are medium), average variance inflation factor (AVIF; acceptable if < 3.3), *R* ^2^ contribution ratio (RCCR; acceptable if > 0.90), and statistical suppression ratio (SSR; acceptable if > 0.70) (Kock 2022). We specified two models. Model one included those assigned to both the intervention and control groups, predicting staying in the study long enough to complete follow‐up measures of behavior in a logistic PLS model. In the second model, we included only the intervention group, predicting the number of intervention sessions they attended during the intervention program.

While the randomized controlled trial from which these data are drawn experienced substantial dropout and attrition, the current study explored engagement and retention as dependent variables. From a statistical perspective, this means that no missing data was observed in the current study, as all participants who completed baseline were included in statistical analyses, with dropout from the trial recorded in and of itself as an outcome metric. Assuming a moderate sized *R* ^*2*^ of 0.20 and *α* = 0.05, the current study required a minimum sample of 65 to achieve a power of 0.80 (Faul et al. 2007).

## 3 Results

Demographic and descriptive information for the sample is presented in Table 1.

**TABLE 1:** Demographic information for the sample predicting intervention retainment and engagement in parent‐child dyads.

Demographics	Total	Retained	Dropout
Parent gender			
Female	95.5%	97.4%	94.0%
Male	3.4%	2.6%	4.0%
Non‐binary	1.1%	0.0%	2.0%
Child gender			
Female	41.3%	45.5%	38.1%
Male	58.7%	54.5%	61.9%
Parental education			
University educated	59.1%	71.1%	50.0%
Not university educated	40.9%	28.9%	50.0%
Parental employment			
Employed	80.7%	81.6%	80.0%
No employed or stay at home parent	19.3%	18.4%	20.0%
Recruitment source			
Social media	44.3%	52.6%	58.0%
Panel company	55.7%	47.4%	42.0%
Autonomous motivation			
Mean	3.76	3.82	3.70
Standard deviation	0.73	0.68	0.76
Number of sessions attended			
Mean	1.40	1.95	0.92
Standard deviation	1.22	1.21	1.02
Attendance per session			
Session 1	50.0%	54.2%	45.8%
Session 2	47.1%	54.5%	30.8%
Session 3	33.3%	54.5%	15.4%
Session 4	14.6%	27.3%	14.6%
Retained in the intervention			
Yes	43.2%	—	—
No	56.8%	—	—

### 3.1 Predicting Study Completion

The logistic model predicting completion of the intervention showed acceptable quality indices (GoF = 0.753, AVIF = 1.15, SPR = 0.857, RSCR = 1.00), predicting a large portion of variance in intervention retention (*R* ^2^ = 0.597, *p* = < 0.001). Those who were retained in the trial were more autonomously motivated (*β* = 0.25, *p* < 0.001, 95% CI [0.05, 0.44]), were more likely to be mothers (*β* = −0.19, *p* = 0.029, 95% CI [−0.39, 0.00]), were more likely to have been recruited from social media as compared to a panel company (*β* = 0.41, *p* < 0.001, 95% CI [0.22, 0.60]), and were more likely to be university educated (*β* = 0.33, *p* < 0.001, 95% CI [0.14, 52]). However, no effects of child gender (*β* = 0.12, *p* = 0.132, 95% CI [−0.09, 0.32]), parental employment (*β* = −0.06, *p* = 0.276, 95% CI [−0.27, 0.14]), or intervention group were observed (*β* = −0.09, *p* = 0.185, 95% CI [−0.30, 0.11]).

### 3.2 Predicting Intervention Engagement

The model predicting engagement in the intervention showed acceptable quality indices (GoF = 0.621, AVIF = 1.12, SPR = 0.83, RSCR = 1.000), predicting a moderate portion of variance in session attendance (*R* ^2^ = 0.406, *p* < 0.001). Autonomous motivation showed a positive non‐zero effect on session attendance (*β* = 0.24, *p* = 0.030, 95% CI [0.00, 0.49]). Attendance was higher when the parent was university educated (*β* = 0.30, *p* = 0.011, 95% CI [0.05, 0.54]), when the parent within the dyad was a father (*β* = 0.23, *p* = 0.040, 95% CI [−0.02, 0.48]), when the child within the dyad was a daughter (*β* = −0.41, *p* < 0.001, 95% CI [−0.65, −0.18]), and but did not vary between dyads recruited from social media or panel companies (*β* = 0.08, *p* = 0.272, 95% CI [−0.18, 0.35]), or between employed and stay‐at‐home or unemployed parents (*β* = 0.02, *p* = 0.432, 95% CI [−0.25, 0.29]).

## 4 Discussion

The current study aimed to identify correlates of intervention retention and engagement using a combination of motivational and socio‐structural variables. The number of sessions attended was predicted by autonomous motivation, parent and child gender, and parental education, while being retained in the intervention was predicted by autonomous motivation, parent gender, parental education, and recruitment source.

Previous evidence has indicated engagement in psychological and behavior change programs is often associated with socio‐structural variables, such as demographics and education. This was partially supported in the current research, as parents reporting more education were more likely to both complete the intervention and to attend more online sessions, in line with previous research (Beatty and Binnion 2016). While speculative, this may be as more educated parents have higher health literacy levels (van der Heide et al. 2013), and thus place greater value on behavior change and active participation in health (McAnally and Hagger 2023). However, more information is required to fully unveil why educated individuals are more likely to engage in health promotion interventions, and by extension, how to engage and retain less educated individuals, particularly given these less educated people are often more likely to report more severe health issues across the lifespan (van der Heide et al. 2013). In contrast, we observed no effects of parental employment on intervention engagement or retention. While this is in line with some other evidence from psychological interventions (Beatty and Binnion 2016), we had expected a potential effect of parental employment given previous research has flagged being busy or having competing demands for time as a key barrier to physical activity in parents (Hamilton and White 2010). However, while previous evidence indicates time concerns is a key issue for parental physical activity, it may be that this does not apply to engaging in physical activity interventions, or other competing uses of time are more impactful than employment (Bellows‐Riecken and Rhodes 2008).

In line with our expectations that women tend to show better completion rates to interventions mothers were more likely to remain in the intervention to complete follow‐up measures. Yet, fathers showed better attendance to sessions than mothers. While these effects should be interpreted with the caveat that our sample comprised mostly of mothers, it is plausible these findings may also be explained by the specifics of the given behavior: physical exercise. For example, while women often show better adherence in research, some research has indicated males have better adherence to physical activity interventions (Vancampfort et al. 2017). This may be particularly pronounced in the current context of parent‐child dyads, where previous research has shown that while mothers are typically more invested in children's educational outcomes, fathers are often more engaged when it comes to children's physical activity and sporting related outcomes (Gathwe et al. 2022; Milošević and Veskovic 2013). Thus, it may be that fathers showed better engagement with the intervention due to their overall interest in sporting outcomes, although whether this is the case requires additional research. However, given the majority of parents who consented to join the study were mothers, such results must be considered with due caution given the small number of fathers may decrease the accuracy or generalizability of results.

Interestingly, we also observed that parents with daughters had better session attendance that parent‐child dyads where the child was male. One plausible explanation for this effect may be a carryover effect due to the fact parents in the sample were predominantly female, as previous research has shown that parents may be more engaged in family physical activity with same‐gender children (Lawler et al. 2022). Thus, the predominantly mother parent group recruited in the current research may have been more engaged, as reflected in better participation in sessions, when the target child in the dyad was also female. Future research may seek to verify this, employing a wider sample with a better balance of mothers and fathers in parent‐child dyads to investigate effects.

Parents who were autonomously motivated to be active were both more likely to complete the intervention than parents lacking autonomous motivation for physical activity and attended more intervention sessions. While measures of autonomous motivation are normally directly linked to the target behavior (i.e., measuring autonomous motivation for physical activity when the outcome behavior is physical activity), this may be expected as those who hold autonomous motives around exercise likely form functional beliefs in favor of being active (Hagger and Chatzisarantis 2014), which may reflect in a willingness to engage in physical activity related behaviors regardless of challenges or other issues (Hein et al. 2021). While this is consistent with previous evidence regarding autonomous motivation predicting more frequent actual physical activity behavior, the current findings extend upon these results indicating autonomous motivation may also promote adherence to physical activity related behavior change programs. Although this finding is not theoretically surprising, it poses concerning questions for motivation‐based interventions ‐ indicating that, at least in the current program which included numerous examples of autonomous supportive techniques for families (Phipps et al. 2024; Ahmadi et al. 2023; Teixeira et al. 2020), those who might benefit most from a program targeting physical activity motivations and beliefs were also the least likely to actively engage in that program. While this requires additional research to confirm this effect, it has clear implications for the wider issue of encouraging inactive families who are not motivated to change.

This is of course not a new problem for health psychologists as almost all active strategies and clinical practices require a degree of willingness to change (Hardcastle et al. 2015). Potential solutions include multi‐level strategies, as advocated in the COM‐B and behavior change wheel framework (Michie et al. 2011). For example, the current results may further highlight the need for more intensive strategies like the program tested in the current study to work in conjunction with wider public health programs which foster readiness to change, such that high‐risk individuals have at least the baseline motivation and abilities needed to attempt to change their behavior. However, while such models have shown promise in more “controlled” environments' like workplaces or schools (Burton et al. 2021; Ojo et al. 2019), they may be more difficult to apply in broader contexts like families or general population cases. Alternatively, it is possible that efficacious behavior change programs may require additional tailoring to meet the needs of those most in need, as per the stage‐based models of behavior change (Prochaska et al. 2009), for example by lowering barriers to entry or focusing on reducing amotivation before transitioning to attempts to build autonomous or intrinsic motivations (Hardcastle et al. 2015; Ma et al. 2021). Future research is needed to elucidate the best path forward in this regard, considering not only the overall effectiveness of intervention strategies, but how they might apply to those most at risk.

Finally, it is interesting to note that while recruitment source was included as a covariate in analyses, and no specific hypotheses were made in this regard, we observed a modest effect of recruitment source on the likelihood of being retained in the intervention program, as those recruited from social media were significantly more likely to remain in the trial than those recruited via a panel company. While this is not an oft discussed element of intervention or longitudinal research, some previous literature has discussed similar potential concerns with participants sourced from opt‐in panel companies compared to crowdsourced participants when comparing data quality metrics like manipulation and attention checks (More et al. 2022; Peer et al. 2015; Zhang and Gearhart 2020). While this effect warrants replication, it raises concerns about whether the convenience and scope of recruitment offered by professional panel companies may need to be considered in light of other concerns when collecting data for interventions and trials.

### 4.1 Limitations

While the current study provides a useful investigation of fidelity in an online psychological intervention, it is inherently not without its limitations. Foremost among these lies in the inherent issue of recruitment for unmotivated samples. That is, while we were able to assess the predictors of intervention fidelity in those who consented to the research, it is likely that those who would most benefit from motivational interventions would also be apathetic toward joining these interventions if they lack motivation to change. Thus, while it is important to assess who engages in interventions after enrollment, the current research is unable to assess the related issues regarding enrollment itself. This is particularly pronounced as study in the current study was predominantly female and well educated. While this is not uncommon in research, it none‐the‐less is a cause for concern that results may not be generalizable to the wider population and again flags the importance and difficulty of recruiting other populations. Secondly, the current study used a correlational prospective design, and we are thus unable to make definitive assertions regarding causality in our model. Thirdly, while we used observed measures of intervention completion and engagement, we do not individually assess the reasons behind non‐compliance in the current study and are indeed inherently unable to provide detailed information on the reasons behind non‐attendance or non‐completion from those participants who withdrew or did not attend sessions without notice. Future research may seek to extend upon the current findings using mixed methods or qualitative designs to tap the reasoning behind participants engagement or lack thereof in intervention programs. Fourthly, when considering demographic information, groups were dummy coded for statistical and pragmatic reasons, for example with stay‐at‐home and currently unemployed parents grouped together as *not working*, or those with bachelors and master level university education grouped together as *university educated*. However, it remains plausible that some differences between grouped categories may exist. Future research with more expansive sample sizes may seek to further investigate these potential differences. Finally, it is also important to consider that as per integrated models of behavior change (Hagger and Chatzisarantis 2014; Phipps et al. 2021), while autonomous motivation is a key driver of behavior, it is by no means likely to account for human behavior in its entirety. For example, as the effects of autonomous motivation on behavior may be modeled via attitudes, norms and control beliefs (Hagger and Chatzisarantis 2014; Phipps et al. 2025), or behavior may occur for reasons independent of autonomous motivation, such as controlled motivations or automatic processes (Phipps et al. 2020; Hagger and Hamilton 2020). Future research with larger sample sizes may seek to expand upon the current findings, investigating a more complete set of motivations and cognitions in predicting intervention engagement and retention.

### 4.2 Conclusions

The current study sought to investigate the role of demographics and autonomous motivation in completing and engaging in an online integrated behavior change theory intervention targeting family physical activity. Autonomously motivated and more educated participants were more likely to both complete the intervention and engage, while fathers were more likely to engage in intervention content, but mothers were more likely to complete intervention measures. Variation in intervention engagement between demographic groups is somewhat in line with previous evidence. However, the higher engagement in the intervention for those already reporting higher levels of autonomous motivation poses an important issue for how to improve physical activity in the highly unmotivated, although this effect requires additional research to unpack.

## Conflicts of Interest

The authors declare no conflicts of interest.

## Data Availability

Supplementary Materials and model outputs may be found at https://osf.io/hcjw9/.
